# A pancancer overview of FBN1, asprosin and its cognate receptor OR4M1 with detailed expression profiling in ovarian cancer

**DOI:** 10.3892/ol.2021.12911

**Published:** 2021-07-09

**Authors:** Rachel Kerslake, Marcia Hall, Paola Vagnarelli, Jeyarooban Jeyaneethi, Harpal S. Randeva, George Pados, Ioannis Kyrou, Emmanouil Karteris

**Affiliations:** 1 Department of Life Sciences, Division of Biosciences, College of Health, Medicine and Life Sciences, Brunel University London, Uxbridge UB8 3PH, UK; 2 Mount Vernon Cancer Centre, Northwood, Middlesex HA6 2RN, UK; 3 Warwickshire Institute for the Study of Diabetes, Endocrinology and Metabolism (WISDEM), University Hospitals Coventry and Warwickshire NHS Trust, Coventry CV2 2DX, UK; 4 Warwick Medical School, University of Warwick, Coventry CV4 7AL, UK; 5 First Department of Obstetrics and Gynaecology, Aristotle University of Thessaloniki, School of Medicine, Thessaloniki 54124, Greece; 6 Centre for Sport, Exercise and Life Sciences, Research Institute for Health and Wellbeing, Coventry University, Coventry CV1 5FB, UK; 7 Aston Medical Research Institute, Aston Medical School, College of Health and Life Sciences, Aston University, Birmingham B4 7ET, UK; 8 Division of Thoracic Surgery, The Royal Brompton and Harefield NHS Foundation Trust, Harefield Hospital, Harefield UB9 6JH, UK

**Keywords:** fibrillin-1, asprosin, cancer, ovarian cancer, olfactory receptor, olfactory receptor 4M1

## Abstract

Ovarian cancer affects >295,000 women worldwide and is the most lethal of gynaecological malignancies. Often diagnosed at a late stage, current research efforts seek to further the molecular understanding of its aetiopathogenesis and the development of novel biomarkers. The present study investigated the expression levels of the glucogenic hormone asprosin [encoded by fibrillin-1 (*FBN1*)], and its cognate receptor, olfactory receptor 4M1 (OR4M1), in ovarian cancer. A blend of *in silico* open access The Cancer Genome Atlas data, as well as *in vitro* reverse transcription-quantitative PCR (RT-qPCR), immunohistochemistry and immunofluorescence data were used. RT-qPCR revealed expression levels of *OR4M1* and *FBN1* in clinical samples and in ovarian cancer cell lines (SKOV-3, PEO1, PEO4 and MDAH-2774), as well as the normal human ovarian surface epithelial cell line (HOSEpiC). Immunohistochemical staining of a tissue microarray was used to identify the expression levels of OR4M1 and asprosin in ovarian cancer samples of varying histological subtype and grade, including clear cell carcinoma, serous ovarian cancer and mucinous adenocarcinoma. Immunofluorescence analysis revealed asprosin expression in SKOV-3 and HOSEpiC cells. These results demonstrated the expression of both asprosin and OR4M1 in normal and malignant human ovarian tissues. This research invokes further investigation to advance the understanding of the role of asprosin and OR4M1 within the ovarian tumour microenvironment.

## Introduction

The tumour microenvironment has received growing interest owing to its role in metabolic dysregulation and tumorigenesis. Recent studies have associated dysregulation of extra cellular matrix (ECM) proteins, such as fibrillin-1, with tumorigenesis. The structural glycoprotein, fibrillin-1, is one of two cleavage products encoded by the *FBN1* gene (1). *FBN1* encodes a 66 exon proprotein known as profibrillin-1, that is proteolytically cleaved within the 65^th^ exon at the consensus sequence X-Arg-X-Lys/Arg-Arg-X by the enzyme furin (1,2). Cleavage produces the 320 kDa glycoprotein fibrillin-1 and the recently discovered 30 kDa glucogenic hormone, asprosin (3).

Asprosin was recently identified by Romere *et al* (3) through an investigation of Neonatal Progeroid Syndrome (NPS); a disorder characterised by reduced insulin despite maintenance of euglycemia, extreme leanness and partial lipodystrophy (4). The pathogenesis of NPS is attributed to premature ablation of profibrillin-1 as a result of a truncation mutation within the *FBN1* gene (3). Investigation of NPS pathophysiology led to the classification of asprosin - the c-terminal cleavage product of profibrillin 1 - as a novel orexigenic and glucogenic hormone, involved in the regulation of glucose homeostasis (3).

Elevated circulating levels of asprosin are present in patients with metabolic syndrome manifestations, such as insulin resistance and type 2 diabetes mellitus (T2DM), and are associated with obesity (5–7). Adipose tissue is the primary source of asprosin secretion, with recent data showing that patients with cancer-related anorexia exhibit significantly lower asprosin plasma levels compared to control counterparts (8,9). There is increasing evidence associating the expression of asprosin with metabolic disorders and complications during pregnancy, such as gestational diabetes, and preeclampsia, as well as intra-uterine growth restriction (10). Additionally, elevated circulating asprosin levels have been noted in women with polycystic ovarian syndrome (PCOS), although further research is required to clarify the relevant role of obesity in this population (11).

Olfactory Receptor 743, an orphan G protein-coupled receptor (GPCR), was recently identified as one of the possible receptors of asprosin in mice, whilst the human ortholog, olfactory receptor 4M1 (OR4M1), is considered to be the primary asprosin receptor in humans (12).

Detection of peripherally expressed olfactory receptors (ORs) is now well-documented; despite initial beliefs for localised expression of these receptors solely within the olfactory epithelium of the nasal cavity (12). Existing data suggest that expression of OLFR734 (and its orthologue OR4M1) may involve the testis, whilst emerging evidence further indicates that expression may also extend to other reproductive tissues, such as the ovaries, with further implications for fertility in mammals (13,14). Recent data present expression of this receptor in the ovaries of murine and bovine samples, supporting an auto/paracrine circuit between asprosin and OR4M1 which may be implicated in female fertility, as well as healthy ovarian follicular function (14). However, expression of OR4M1 is yet to be explored in human tissues past the testis, with the exception of peripheral blood mononuclear cell expression in cases of traumatic brain injury (15).

Ovarian cancer is one of the most lethal gynaecological malignancies, affecting over 295,000 women worldwide (16). Dysregulation of *FBN1* [which is expressed within the theca interna and stroma of healthy ovarian tissue (17)], in ovarian cancer, through Aurora A and BRCA 2 signalling, is associated with invasion and metastasis of tumour cells (18). Moreover, *FBN1* is linked with worse overall survival, as well as advanced stage of disease in high grade serous ovarian cancer (19). However, studies have yet to investigate the expression of asprosin in reproductive tissues in both healthy women and those with ovarian cancer.

The regulation of glucose metabolism in ovarian cancer has been studied extensively, however, certain mechanisms are not fully elucidated. For example, hyperglycaemia drives ovarian tumour growth independently of insulin status (20). Of note, this heightened state of glucose metabolism is thought to accelerate tumour growth through increased aerobic glycolysis in what is known as the ‘Warburg effect’, and leads to a worse prognosis in cancer, including ovarian cancer (21). Increased expression of the glucose transporter GLUT-1 in ovarian cancer is also linked to a decrease in overall survival, suggesting that glucose abundance is a rate limiting factor of glucose metabolism (22). In this context, investigating the expression of both *FBN1* and the novel glucogenic hormone asprosin in human ovarian tissues will enhance our understanding of the underlying molecular mechanisms implicated in ovarian cancer, as well as the regulation of its tumour microenvironment (20).

In this study -apart from the *in silico FBN1* pan-cancer expression- we provide novel evidence of the protein expression of asprosin in ovarian cancer patients and healthy controls. We also demonstrate -to the best of our knowledge- for the first time expression of the olfactory receptor OR4M1 in the same tissues, raising the prospect of an auto/paracrine regulation at the ovarian level. Finally, we mapped the cellular distribution of asprosin in human ovarian cell lines, as well as the expression of the cognate receptor OR4M1.

## Materials and methods

### 

#### Bioinformatics analysis

A Pancancer set of TCGA data was downloaded through cBioPortal (www.cbioportal.org) and Shiny Methylation Analysis Tool (SMART) (www.bioinfo-zs.com/smartapp/). Expression was validated through GEPIA (gepia.cancer-pku.cn/) and GTeX (gtexportal.org/home/). Survival plots were obtained using Kaplan-Meier Plotter [www.kmplot.com; (23)]. TCGA data sets are described under abbreviations.

#### Cell culture

SKOV-3 (ECAAC 91091004), PEO1 (ECAAC 10032308), PEO4 (ECAAC 10032309) and MDAH-2774 (ATCC CRL-10303) ovarian cancer cells were cultured using aseptic technique and incubated at 37°C in humidified conditions at 5% CO_2_. Cells were regularly sub-cultured at 80% confluency in T75 filter head flasks (Thermo Fisher Scientific, Inc.). SKOV-3 and MDAH-2774 were cultured in Dulbecco's modified Eagle's medium (DMEM) (Thermo Fisher Scientific, Inc.). PEO1 and PEO4 were cultured in Roswell Park Memorial Institute (RPMI) (Thermo Fisher Scientific, Inc.). Media were supplemented with 10% foetal bovine serum (FBS) and 1% penicillin-streptomycin (Thermo Fisher Scientific, Inc.). Normal ovarian epithelial cells, HOSEpiC (cat. no. 7310) were cultured in Poly-L-Lysine coated flasks (5 µg/ml) according to the protocol provided by the supplier (ScienCell), with Ovarian Epithelial Cell Medium (OEpICM) supplemented with 1% Ovarian Cell Growth Supplement and 1% penicillin-streptomycin (ScienCell) and 10% FBS (Thermo Fisher Scientific, Inc.). For disassociation of adherent cells, TrypLE express (Thermo Fisher Scientific, Inc.) was used. Cell count and viability were detected manually using a Neubauer Counting chamber with trypan blue (Invitrogen; Thermo Fisher Scientific, Inc.) exclusion method. SKOV-3 were derived from a human epithelial ovarian cancer patient and are haplo-diploid adherent cells that carry a P53 mutation. PEO1 are derived from human ovarian adenocarcinoma. PEO4 were derived from the same patient as PEO1 although were harvested following treatment with platinum-based chemotherapeutics and are cisplatin resistant. MDAH-2774 were derived from a patient with ovarian endometroid adenocarcinoma. The primary cell line, Human Ovarian Surface Epithelial cells (HOSEpiC), referred to as ovarian surface epithelial cells (OSE), were obtained commercially at passage 1 and are classified as normal ovarian epithelial cells.

#### Clinical ovarian samples

Clinical ovarian cancer samples (n=12, Table I) and samples from healthy volunteers (n=6, Table II) were obtained from patients at the First Department of Obstetrics and Gynaecology, ‘Papageorgiou’ General Hospital, Medical School, Aristotle University, Thessaloniki, Greece. Specimens from the 12 (average, 61.8 years; range, 48–75) patients with ovarian cancer were taken during laparotomy for debunking surgery. Furthermore, in 6 reproductive-age women (average, 41.7 years; range, 39–45) without any ovarian pathology who had completed their reproductive cycle and underwent laparoscopic myomectomy for leiomyomas during the follicular phase of the cycle, an ovarian sample was taken. Institutional ethical approval was provided, and informed consent was obtained from each patient before the collection of samples (Reference: 14/11/STF/06).

#### Immunofluorescence

Cells were washed in phosphate buffered saline (PBS) solution (Thermo Fisher Scientific, Inc.), fixed with ice cold methanol, and washed three times with PBS. Samples were blocked with 5% bovine serum albumin (BSA) buffer (Thermo Fisher Scientific, Inc.), covered with parafilm, and left to incubate for 40 min at 37°C. Asprosin (BioLegend) and OR4M1 (Novus Biologicals) primary antibodies (1:200/1:100 in 5% BSA) were added before incubation at 37°C for 1 h (asprosin) or room temperature overnight (OR4M1). The coverslips were washed three times with PBS before the addition of secondary Alexa Flour 488 antibody (Merck Millipore) at a concentration of 1:200. The samples were covered with parafilm and placed in a humidified chamber for 30 min at room temperature, before being washed three times with PBS. Coverslips were transferred to a glass slide and sealed with a drop of Molecular ProbesProLong Diamond Antifade Mountant with DAPI (Thermo Fisher Scientific, Inc.) and clear nail varnish. The slides were then analysed, and images captured using a DM4000 microscope (Leica) lens at ×100 magnification.

#### Immunohistochemistry of tissue microarray

Paraffin-embedded ovarian tissue microarray slides were purchased from US Biomax Inc. (cat. no. BC11115c). All tissue samples were collected under Health Insurance Portability and Accountability Act (HIPAA) approved protocols, following the appropriate ethical standards with the donors being fully informed and with their consent. Slides comprised of 100 biopsy cores of ovarian tissue: malignant and adjacent (Table SI). Slides were deparaffinised and rehydrated, followed by antigen retrieval using sodium citrate solution (10 mM Sodium citrate in dH_2_O, 0.05% Tween-20, pH 6.0). They were then washed in 0.025% Triton-X in PBS (Thermo Fisher Scientific, Inc.) before a 15-min incubation in 3% H_2_O_2_ followed by additional washes in 0.025% Triton-X in PBS. The slides were blocked with 5% BSA in PBS, followed by incubation with Asprosin/OR4M1 Antibody (1:200/1:100) overnight in a humidity chamber at 4°C.

Slides were then washed three times in 0.025% Triton-X in PBS and incubated with a secondary antibody in 1% rabbit serum (ZytoChem Plus HRP-DAB Kit, Zytomed Systems GmbH) for 1 h. The slides were then washed with 0.025% Triton-X in PBS to ensure the removal of unbound secondary antibody. Then streptavidin-HRP conjugate was added to the bound secondary antibody and the slide incubated for a further 30 min within the humidity chamber. Slides were washed with PBS before the addition of DAB stain. These were then counterstained with haematoxylin and washed with 0.1% sodium bicarbonate. Finally, slides were dehydrated before the addition of DPX and coverslips, then left to dry overnight. Immunoreactivity was analysed using a light microscope (Zeiss GmbH). Results were calculated by two independent reviewers using a percentage score of positive tumour cells, as described previously (24).

#### RNA isolation, cDNA synthesis and reverse transcription-quantitative PCR (RT-qPCR)

Total RNA was extracted from cell lysates using the RNeasy Mini Kit (Qiagen, Inc.), before being reverse transcribed using a cDNA reverse transcription Kit (Applied Biosystems; Thermo Fisher Scientific, Inc.). Sample purity was assessed using Nano-Drop 2000C (Thermo Fisher Scientific, Inc.) and relative gene expression measured using SYBR Green PCR Master Mix (Bio-Rad) and qPCR with a Bio-Rad CFX96 system according to the following conditions (Table III).

*FBN1* primers were designed according to the Harvard Primer bank, whereas *OR4M1* were generated according to a 2013 study (15). Additional primers include the housekeeping gene *YWHAZ* (Table IV). RQ values were calculated as previously described (24), according to the comparative 2^−ΔΔCq^ analysis method (25).

#### Statistical analysis

Statistical analyses were performed using GraphPad prism9^®^ software (GraphPad Software, Inc.). Error bars in graphs are presented as standard error of the mean (SEM). Mann Whitney U test and a one-way ANOVA (Analysis of variance) with Tukey's multiple comparison post hoc statistical tests were applied to the observed measurements from the data. Variances in survival were generated using Kaplan-Meier curves with log-rank test. Beta values were calculated using the SMART methylation tool (SMART). The method for differential analysis conducted by GEPIA is listed as a one-way ANOVA, where disease state (Tumour or Normal) is used as a variable for calculating differential expression: Gene expression against disease state. The expression data are first log2(TPM+1) transformed for differential analysis and the log2FC is defined as median (Tumour) - median (Normal). Genes with higher |log2FC| values and lower q values than pre-set thresholds are considered differentially expressed genes. More information can be accessed at http://gepia.cancer-pku.cn/help.html. Unless stated otherwise, significance was set at P-value <0.05.

## Results

### 

#### Expression of FBN1 in normal tissues

**Figure 1.**
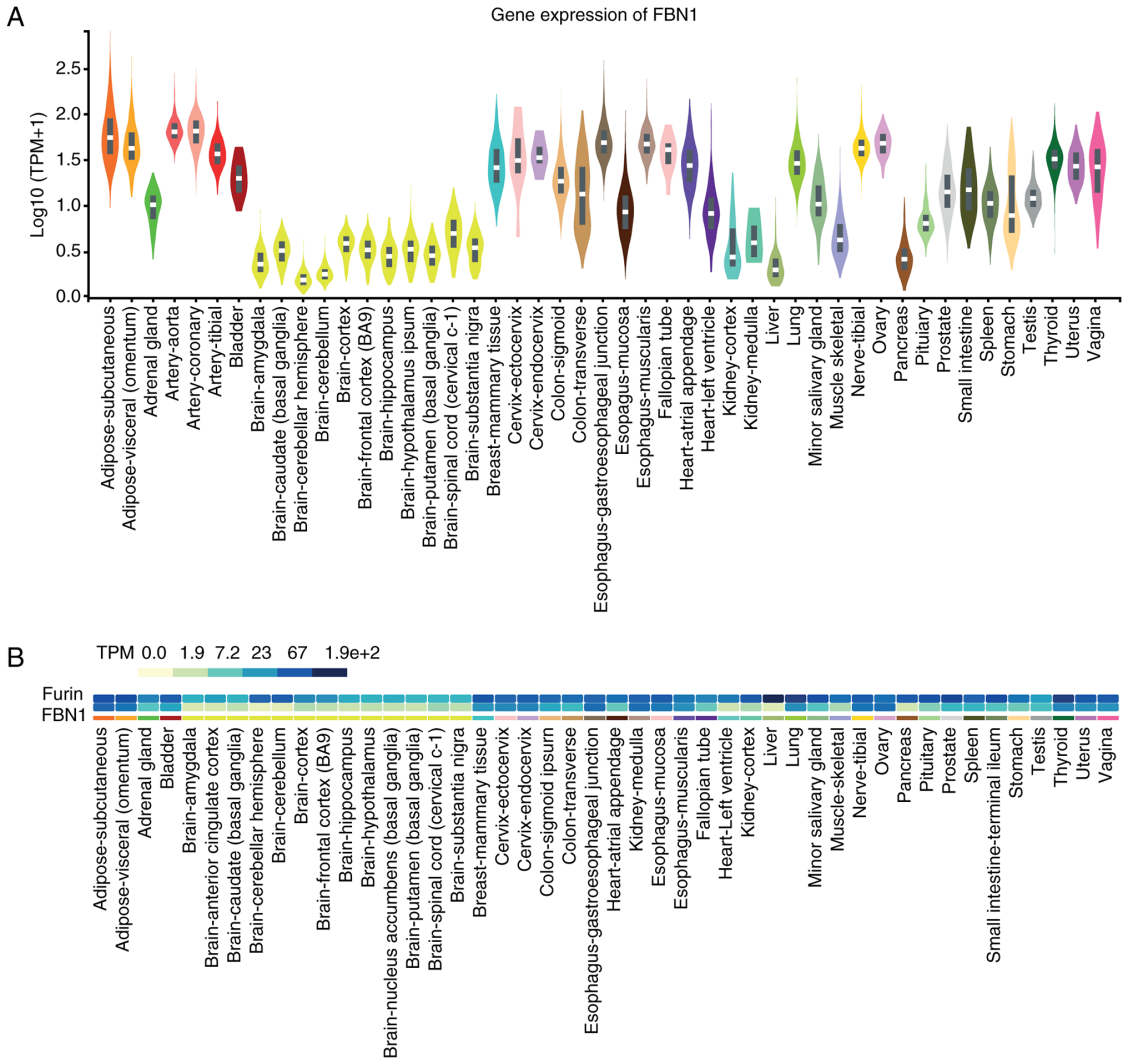
Gene expression of FBN1 in normal tissues. (A) Expression levels of FBN1 in normal human tissues based on available data from The Genotype Tissue Expression project. (B) Co-expression of FBN1 and furin, the enzyme which proteolytically cleaves profibrillin-1 to fibrillin-1 and asprosin, in normal human tissues. The different colours adjacent to furin and FBN1 denote the expression levels of both genes in (B). The darker the colour (dark blue) the higher the expression and the lighter (yellow) the lower the expression (indicated as TPM). The fine different coloured lines underneath the co-expression data in (B) are used for identification purposes and relate to the different coloured violin plots in A. FBN1, fibrillin-1; TPM, transcripts per million.

Initial analyses of *FBN1* expression were conducted using publicly available data from The Genotype Tissue Expression (GTEX) project (Fig. 1). Fibroblasts, arteries, adipose tissue (subcutaneous and visceral) and the ovaries are amongst the tissues that express relatively high levels of *FBN1*, as do the studied female reproductive tissues. Brain and whole blood express the lowest *FBN1* levels, along with the liver and pancreas (Fig. 1A). In the same dataset, we further analysed the co-expression of *FBN1* with the proteolytic enzyme *furin*, which may provide an oversight of potential furin-mediated cleavage release of asprosin in these tissues (Fig. 1B). *Furin* is shown to exhibit ubiquitous expression throughout the human body with high levels detected across all tissues, including those with high *FBN1* expression (e.g., normal human reproductive tissues, such as testis, vagina, uterus and ovaries).

#### Pancancer mapping of FBN1

**Figure 2.**
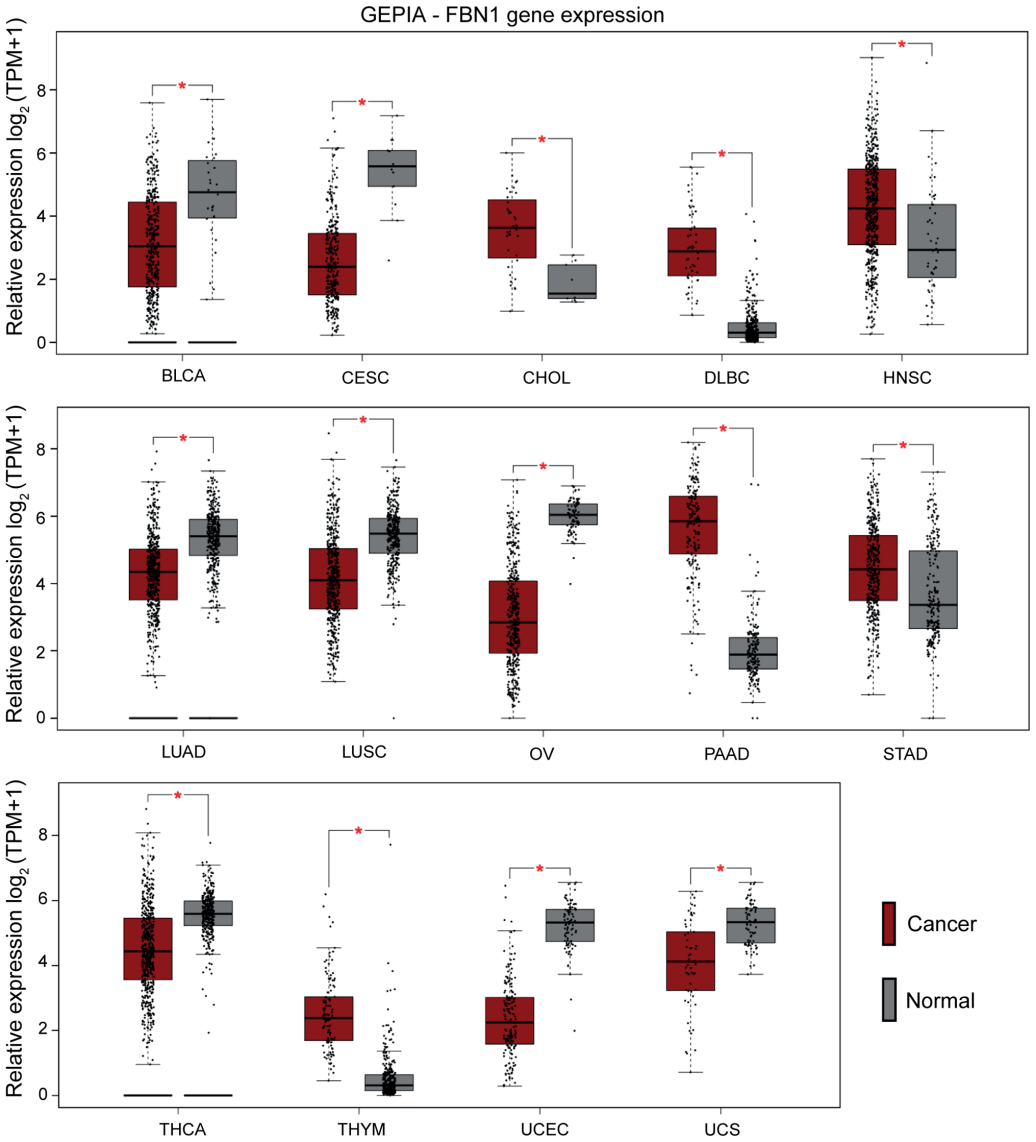
Pancancer profiling of FBN1 expression. Cancer types with significant differences compared with normal tissues (*P<0.01) are presented in the graphs [cancer (red) and normal (grey)]. Cancers with lower expression levels of FBN1 compared with normal samples included UCS, UCEC, THCA, OV, LUSC, LUAD, CESC and BLCA. Those with higher FBN1 expression levels included THYM, STAD, PAAD, HNSC, DLBC and CHOL. BLCA, bladder urothelial carcinoma; CESC, cervical squamous cell carcinoma and endocervical adenocarcinoma; CHOL, cholangiocarcinoma; DLBC, lymphoid neoplasm diffuse large b-cell lymphoma; FBN1, fibrillin-1; GEPIA, Gene Expression Profiling Interactive Analysis; HNSC, head and neck squamous cell carcinoma; LUAD, lung adenocarcinoma; LUSC, lung squamous cell carcinoma; OV, ovarian serous cystadenocarcinoma; PAAD, pancreatic adenocarcinoma; STAD, stomach adenocarcinoma; THCA, thyroid carcinoma; THYM, thymoma; TPM, transcripts per million; UCEC, uterine corpus endometrial carcinoma; UCS, uterine carcinosarcoma.

We expanded our observations by assessing the expression of *FBN1* across 33 different cancer types using TCGA datasets through GEPIA. As presented in Fig. 2, significant differential regulation of FBN1 is noted for the following cancer types: bladder urothelial carcinoma (BLCA), cervical squamous cell carcinoma and endocervical adenocarcinoma (CESC), cholangiocarcinoma (CHOL), lymphoid neoplasm diffuse large B-cell lymphoma (DLBC), head neck and squamous cell carcinoma (HNSC), lung adenocarcinoma (LUAD), lung squamous cell carcinoma (LUSC), ovarian serous cystadenocarcinoma (OV), pancreatic adenocarcinoma (PAAD), stomach adenocarcinoma (STAD), thyroid carcinoma (THCA), thymoma (THYM), uterine corpus endometrial carcinoma (UCEC), and uterine carcinosarcoma (UCS). Of the presented cancers, the female reproductive tissues: uterine, cervical, and ovarian exhibit lower *FBN1* expression compared to corresponding normal tissues.

Given that the methylation status of *FBN1* is of known biomarker potential (26), the FBN1 methylation status for the above cancers was assessed in the same dataset using SMART (Fig. S1). FBN1 methylation in colon adenocarcinoma (COAD) is significantly higher than healthy colon. Similar results are noted for breast (BRCA) and uterine endometrial carcinoma (UCEC). The methylation status of FBN1 within the ovarian cancer data set appears to be highly variable compared to other cancers, as indicated by the beta value of ~0.5; however, there is a lack of comparable normal data for ovarian cancer from TCGA.

**Figure 3.**
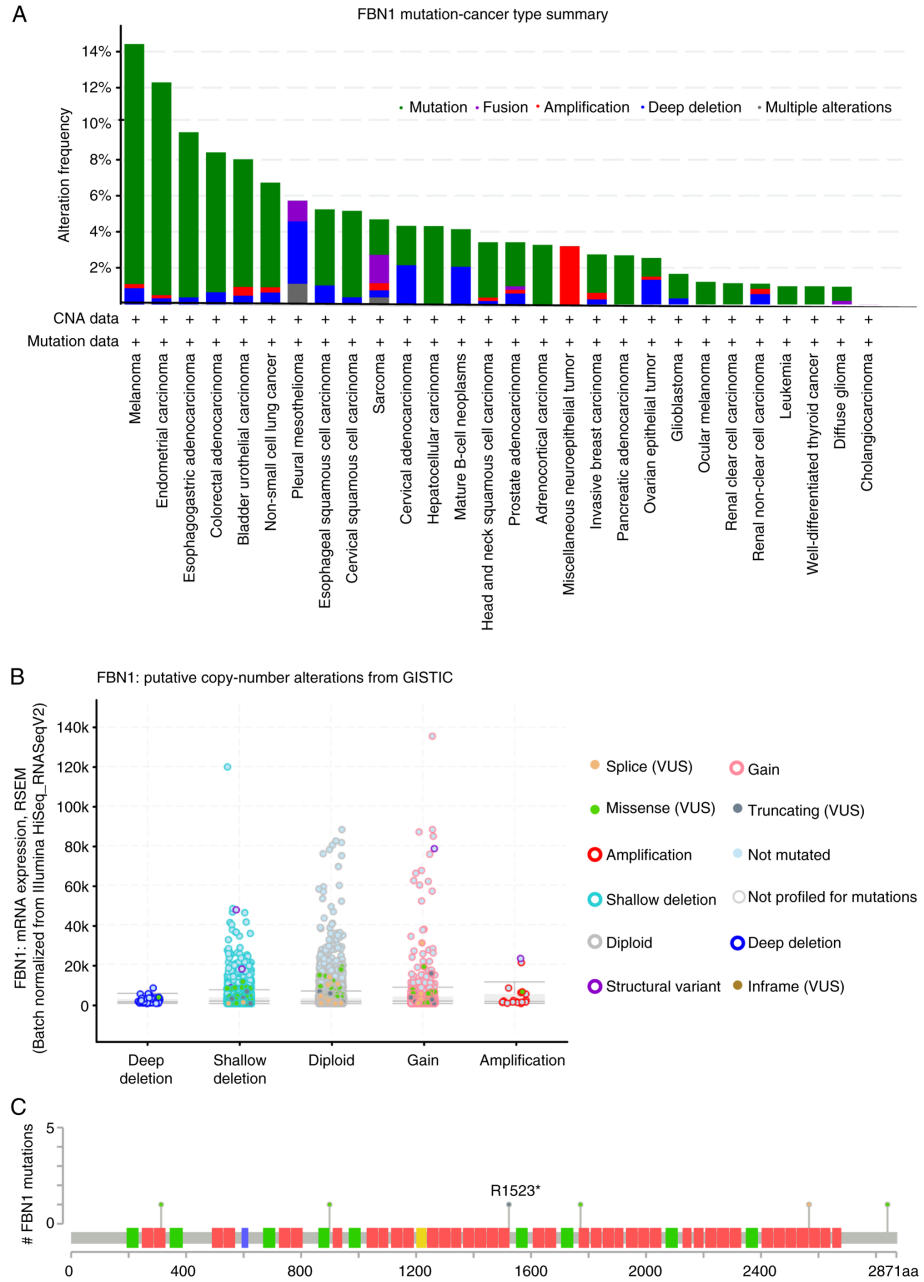
Mutational profile of FBN1. (A) Pancancer overview of the frequency of FBN1 mutations. (B) Copy number of FBN1 alterations across all cancer types (as in Fig. 1A). (C) Location of FBN1 mutations, each lollipop represents an ovarian cancer patient and the corresponding location of the mutation within the gene (Ch15q21.1). Missense mutations are presented as green lollipops, nonsense mutations as black lollipops and splice as orange (source, cBioPortal). CNA, copy number alteration; GISTIC, Genomic Identification of Significant Targets in Cancer; FBN1, fibrillin-1; VUS, variants of unknown significance; RSEM, RNA sequencing by expectation-maximization.

Additional insight was sought through the analysis of *FBN1* using cBioPortal. Mutations of FBN1 within the pancancer cohort of TCGA cancers appear to be most frequent in melanoma, uterine, stomach and colorectal cancer (Fig. 3A). A relatively lower frequency of alterations were detected in ovarian cancer samples compared to the other types of cancer, however, the high percentage of deep deletion within the cases presented must be noted.

Gain of function, shallow deletion and diploid appear to show the highest frequency of copy number variation within the samples (Fig. 3B). Six mutations on the *FBN1* gene were identified in cases of serous ovarian cancer (Fig. 3C). Nonsense and splice-site mutations (black and orange lollipops) give rise to a truncated FBN1-encoded protein, whereas the four missense mutations (green lollipops) cause an amino acid substitution. Of note, one mutation has been identified in the asprosin coding region leading to a lysine to arginine (K2840R) substitution.

#### Expression of FBN1, asprosin and OR4M1 in ovarian cancer

**Figure 4.**
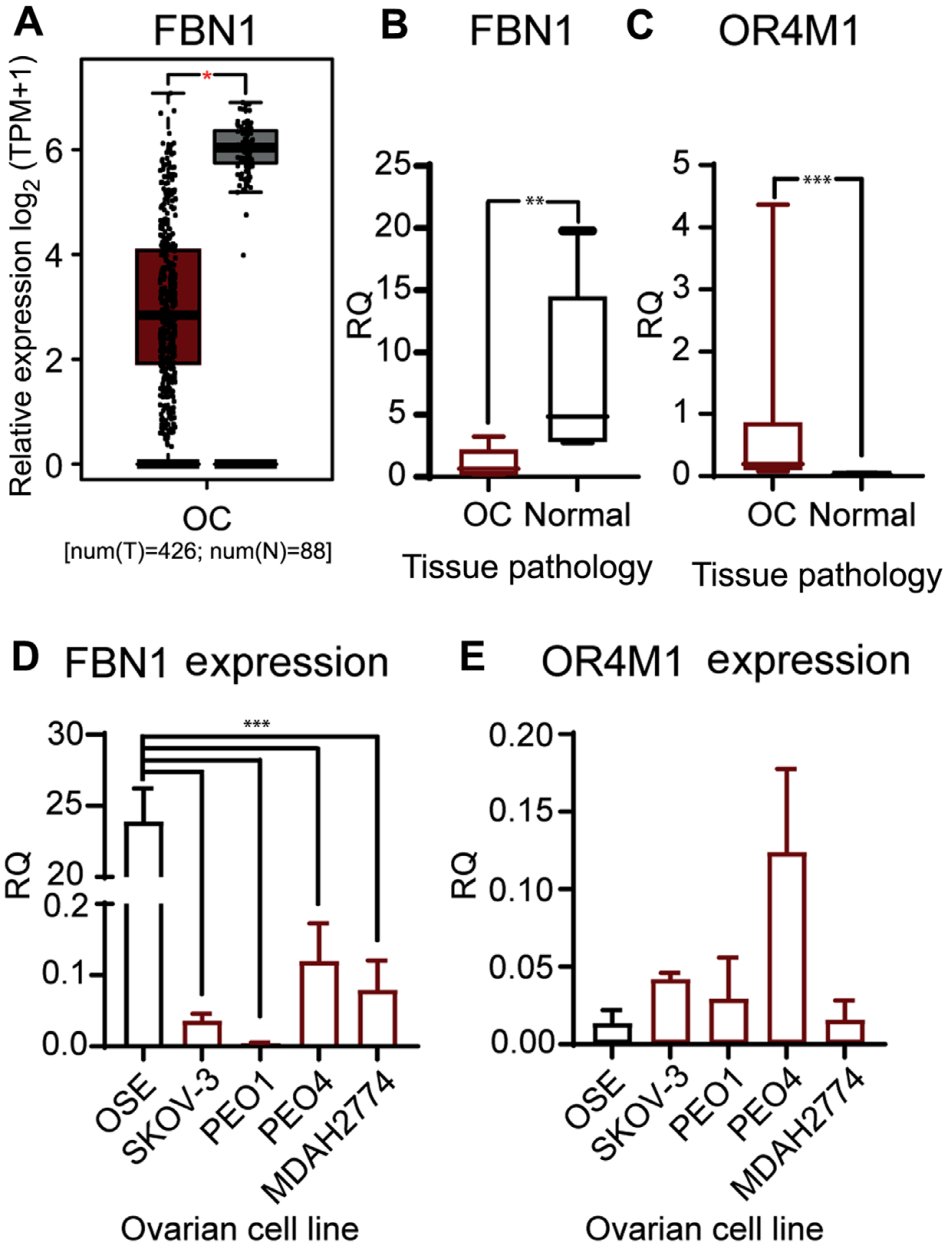
Gene expression of FBN1 and OR4M1 at the ovarian level. (A) Expression data of FBN1 in OC from GEPIA for use as comparison. *P<0.05. Relative expression levels of FBN1 and OR4M1 in OC (red) and normal ovarian tissues (grey) were determined using reverse transcription-quantitative PCR. (B) Significantly lower expression levels of FBN1 in OC samples (OC, n=12; stage III and IV) compared with FBN1 expression in normal ovarian tissue samples from healthy volunteers (n=6). **P<0.001 (samples obtained for the present study; different from the GEPIA cohort in A). (C) Significantly higher expression levels of OR4M1 in OC samples (OC, n=12; stage III and IV) compared with OR4M1 expression in normal ovarian tissue samples from healthy volunteers (n=6). ***P<0.0001. (D) Higher relative expression levels of FBN1 in normal ovarian surface epithelial cells (OSE), and lower expression levels in the studied human ovarian cancer cell lines (SKOV-3, PEO1, PEO4 and MDAH-2774). ***P<0.0001. (E) Lower relative expression levels of OR4M1 in normal ovarian epithelial cells, as well as in the PEO1 and MDAH-2774 human ovarian cancer cell lines, compared with the relatively higher OR4M1 expression noted in SKOV-3 and PEO4 cells. RQ indicates relative change in fold expression to the calibrator gene YWHAZ. FBN1, fibrillin-1; GEPIA, Gene Expression Profiling Interactive Analysis; OC, ovarian cancer; OR4M1, olfactory receptor 4M1; OSE, HOSEpiC cells; TPM, transcripts per million; num(T), number of patients for tumour group; num(N), number of patients for normal group; RQ, relative quantity.

We have validated the in-silico data from TCGA and GTEX (Fig. 4A), using a smaller cohort of patients with ovarian cancer (n=12; stage III and IV). Our data corroborates the previous findings, as it demonstrates that the mRNA expression of *FBN1* was significantly lower in patients with ovarian cancer compared to healthy volunteers (n=6; Fig. 4B). In addition, *OR4M1* expression was significantly up regulated in the same ovarian cancer samples (n=12) compared to the controls (n=6; Fig. 4C). We then measured expression of FBN1 and OR4M1 in five ovarian cell lines: one normal ovarian epithelial cell line (HOSEpiC), and four ovarian cancer cell lines namely, SKOV-3, PEO1, PEO4 and MDAH-2774. FBN1 was significantly over-expressed in HOSEpiC cells compared to all studied ovarian cancer cell lines (Fig. 4D), whereas no apparent change in the expression of OR4M1 was noted across all five cell lines (Fig. 4E).

**Figure 5.**
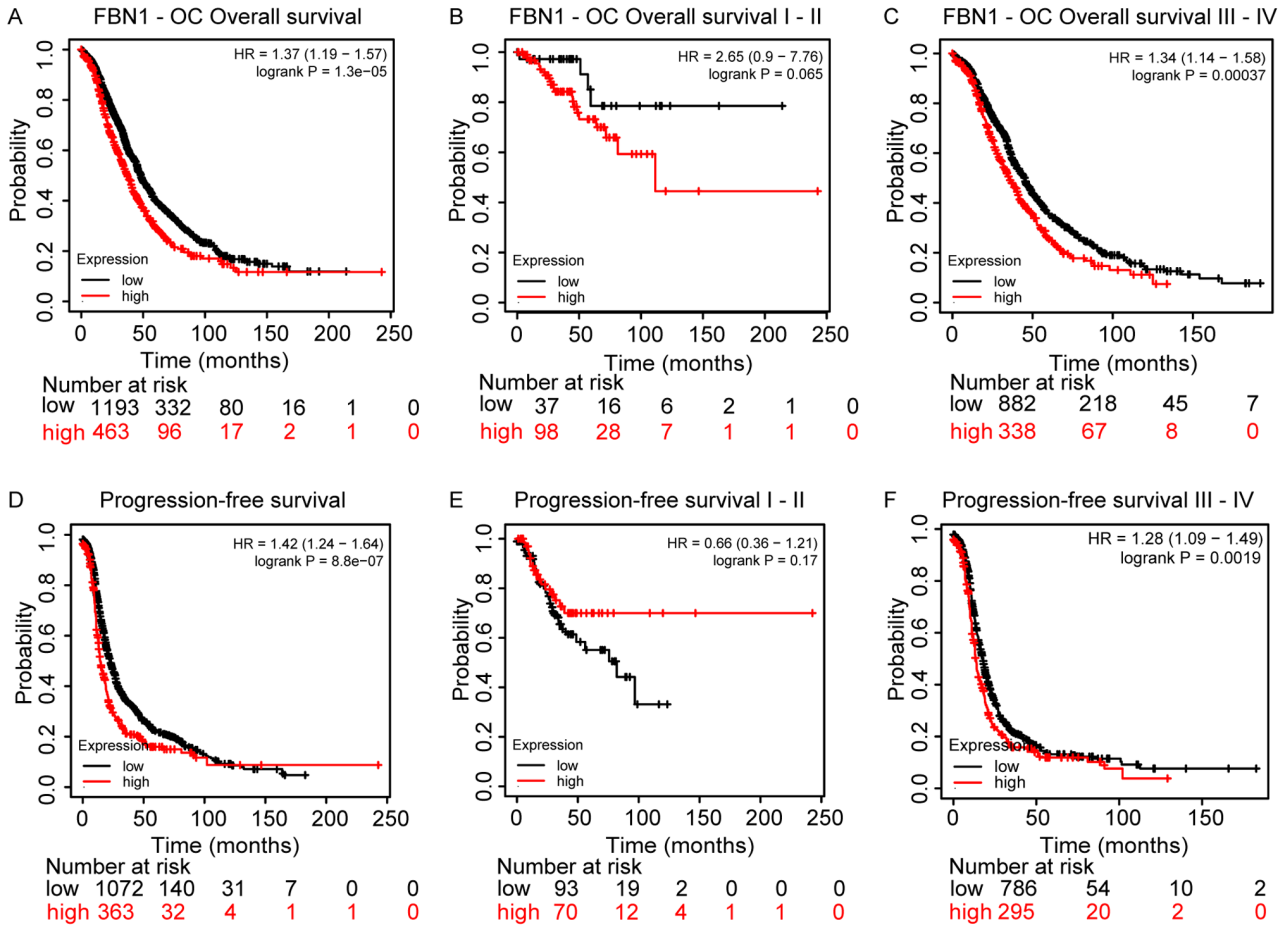
Kaplan-Meier plots revealing the prognostic effects of FBN1 expression in OC. (A) OS in OC. (B) OS in early-stage (I and II) OC. (C) OS in late-stage (III and IV) OC. (D) PFS in OC. (E) PFS in early-stage (I and II) OC. (F) PFS in late-stage (III and IV) OC. FBN1, fibrillin-1; HR, hazard ratio; OC, ovarian cancer; OS, overall survival; PFS, progression-free survival.

Since FBN1 is differentially regulated in ovarian cancer, its prognostic value was also assessed using Kaplan-Meier plots for overall survival (OS) and progression free survival (PFS), Fig 5. Higher *FBN1* expression was associated with poor OS and PFS, Fig. 5A and D, respectively. This predictive power of FBN1 appears to be significant for patients with late stage ovarian cancer (i.e., III and IV), rather than early stage (i.e., I and II), Fig. 5B and C and E and F for OS and PFS, respectively.

**Figure 6.**
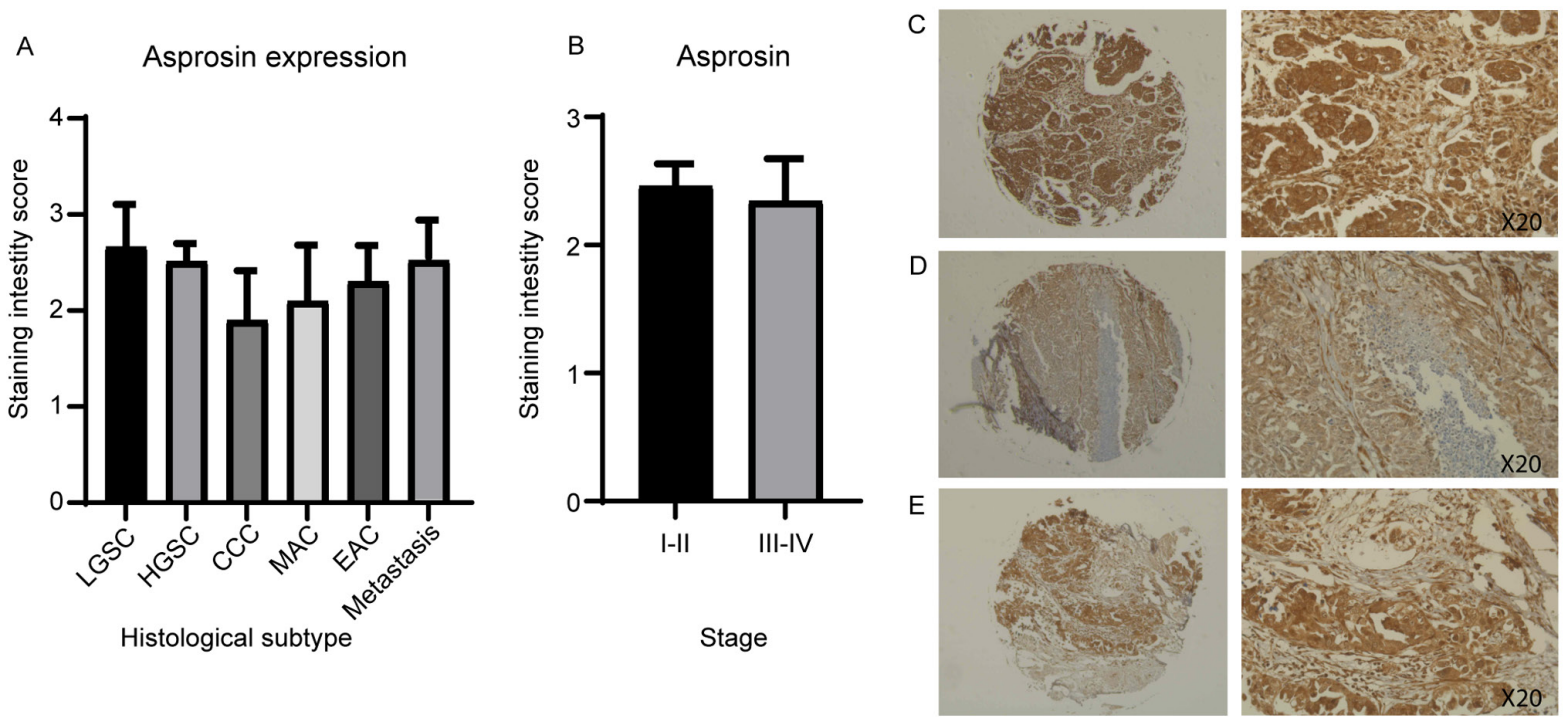
Ovarian tissue microarray, including 90 ovarian cancer cores, stained with asprosin antibody (1:200). Corresponding values for scoring: 0, <10%; 1, 10–25%; 2, 26–50%; 3, 51–75%; and 4, >76% of cells stained. (A) Asprosin staining by histological subtype/grade: LGSC, HGSC, MAC, EAC and CCC. (B) Asprosin staining of early (I and II) and late (III and IV) ovarian cancer stages, revealing no significant difference. (C) HGSC, stage II at ×5 (left) and ×20 (right) magnification. (D) HGSC, stage I at ×5 (left) and ×20 (right) magnification. (E) HGSC, stage III at ×5 (left) and ×20 (right) magnification. CCC, clear cell carcinoma; EAC, endometroid adenocarcinoma; HGSC, high grade serous carcinoma; LGSC, low grade serous carcinoma; MAC, mucinous adenocarcinoma.

**Figure 7.**
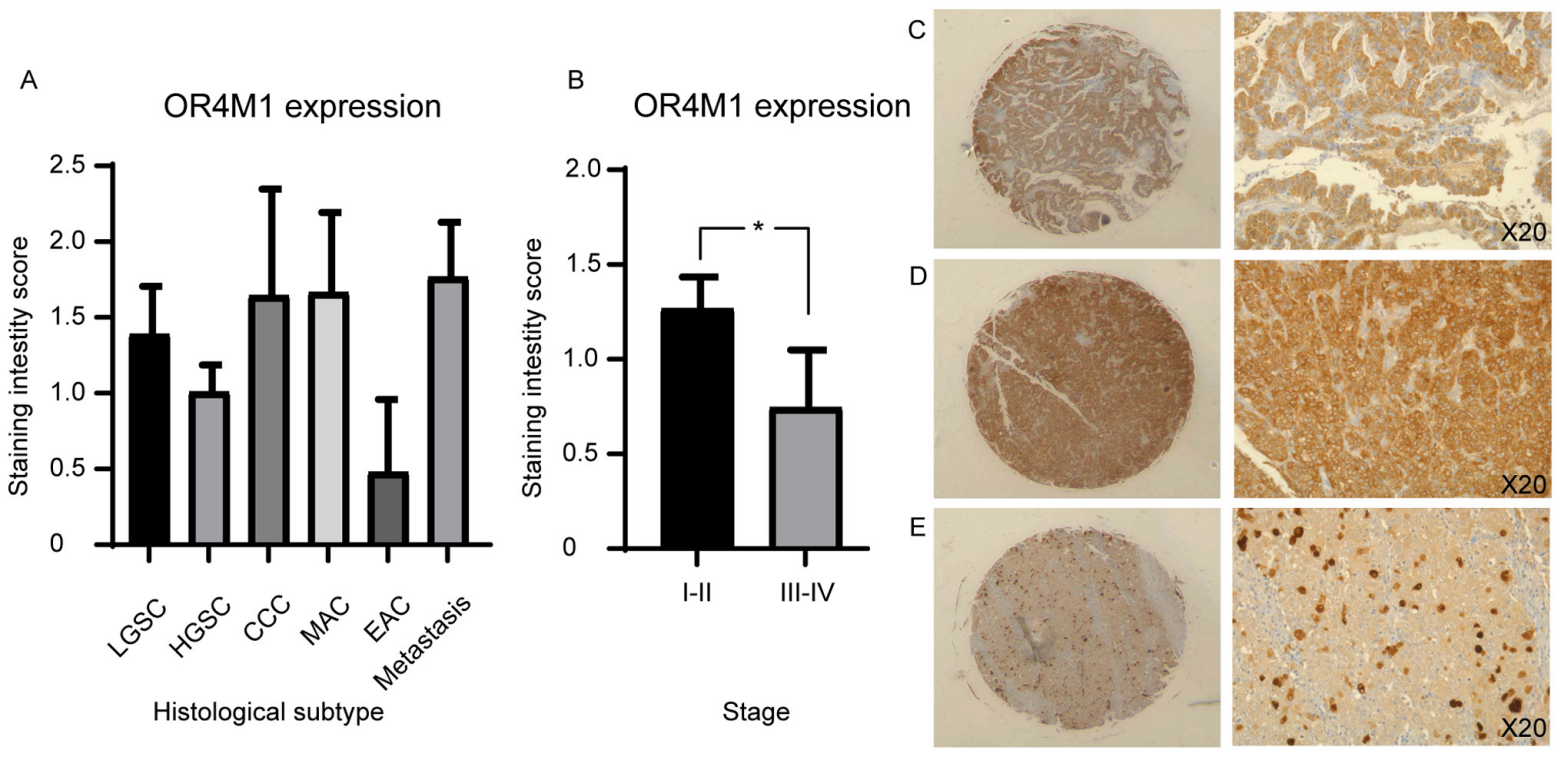
Immunohistochemical staining of an ovarian tissue microarray, including 90 ovarian cancer cores, with OR4M1 antibody (1:100). (A) OR4M1 staining by histological subtype/grade: LGSC, HGSC, MAC, EAC and CCC. (B) Higher OR4M1 staining in early (I and II) compared with late (III and IV) ovarian cancer stages. *P=0.04. (C) HGSC, stage II at ×5 (left) and ×20 (right) magnification. (D) HGSC, stage I at ×5 (left) and ×20 (right) magnification. (E) HGSC, stage III at ×5 (left) and ×20 (right) magnification. CCC, clear cell carcinoma; EAC, endometroid adenocarcinoma; HGSC, high grade serous carcinoma; LGSC, low grade serous carcinoma; MAC, mucinous adenocarcinoma; NAT, normal adjacent tissue; OR4M1, olfactory receptor 4M1.

Immunohistochemical analysis of a tissue microarray containing 90 ovarian cancer cores and 10 normal adjacent tissue (NAT) cores, each representing a different clinical case, was used to measure the protein expression of asprosin and OR4M1 (Figs. 6 and 7). Asprosin was aberrantly expressed across all different histological subtypes (Fig. 6A), with no stage-specific variation when samples were grouped to early (I and II) and late (III and IV) ovarian cancer stages (Fig. 6B). Examination of OR4M1 protein expression revealed similar non-specific expression across different histological subtypes (Fig. 7A). However, higher expression was detected in early (I and II) compared to late (III and IV) ovarian cancer stages (Fig. 7B).

**Figure 8.**
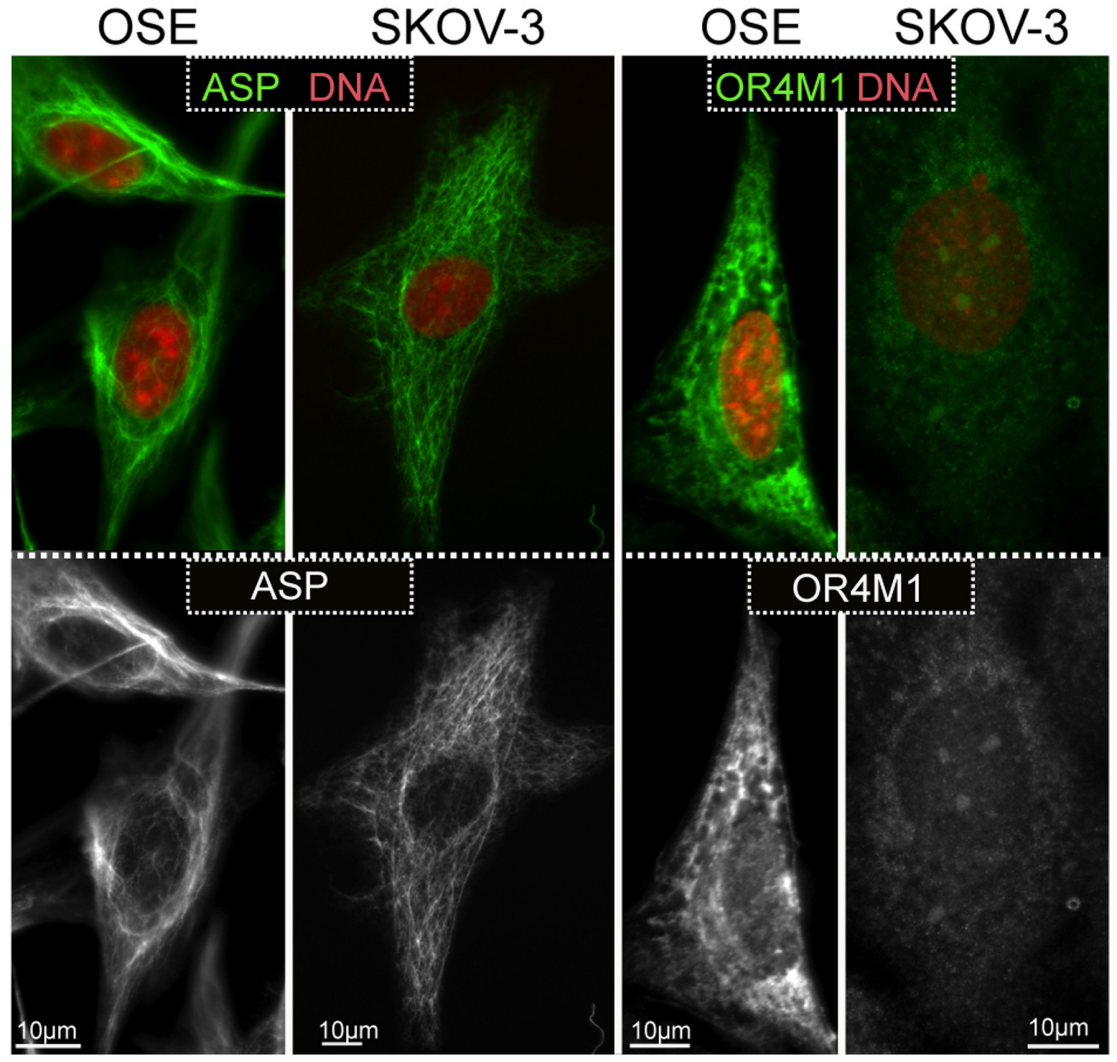
Immunofluorescence imaging of OSE normal human ovarian epithelial cells and SKOV-3 human serous ovarian cancer cells, with DAPI nuclear staining (red) and with ASP and OR4M1 (green). Magnification, ×100 using a Leica DM4000 microscope (Scale bar, 10 µm). ASP, asprosin; OR4M1, olfactory receptor 4M1.

Observations on the expression of asprosin and OR4M1 were expanded using the SKOV-3 ovarian cancer cell line, as well as the normal human ovarian epithelial cell line, HOSEpiC (OSE). Similarly, to the tissue sections, asprosin exhibited a cytoplasmic distribution (associated with structures resembling microtubules or cytoskeleton), whereas OR4M1 appears to be expressed on the plasma membrane and cytoplasm in accordance with the expected distribution of a GPCR (Fig. 8).

## Discussion

This study presents novel data regarding the expression of *FBN1* (the gene encoding profibrillin-1), asprosin (the novel orexigenic/glucogenic hormone which is cleaved from profibrillin-1), and OR4M1 (the human cognate receptor of asprosin) in cancer, focusing on ovarian cancer. Using an in-silico approach, we demonstrate that FBN1 expression is ubiquitous in normal tissues, with high levels seen in fibrous tissues (e.g. in fibroblast cells) and arteries, in addition to female reproductive tissues, such as the uterus and ovaries. Being the main source for the production of circulating asprosin (9), adipose tissue also exhibited high FBN1 expression. To date, asprosin production is thought to be specific to adipose tissue. However, the noted co-expression of FBN1 with the proteolytic enzyme furin in human tissues is indicative of potential production and release of asprosin from other peripheral tissues, such as the ovaries.

Although multiple studies have shown FBN1 mutations as the cause of Marfan syndrome (MFS), which is further associated with increased risk of tumourigenesis (27), very little is known about the role of FBN1 mutations in cancer. Analysis of over one million cancer cases, including stomach, liver, oesophagus, prostate, gynaecological and other cancers, in a national cohort of patients with MFS in Taiwan showed a higher risk of developing cancer in these patients (27). Of note, the data presented from cBioportal in our study, indicate that six FBN1 mutations were present in patients with ovarian cancer, with one of the missense mutations located in the coding region for asprosin. Future GWAS studies are required to explore the potential involvement of these mutations in ovarian cancer.

The presented data from GEPIA in this study, show differential FBN1 expression in 14 cancers, with higher expression noted in cancers of the stomach (STAD) and pancreas (PAAD). The latter is in line with previous research associating increased FBN1 expression in pancreatic islets with cellular progression from hyperplastic to angiogenic to insulinoma (28). Lower FBN1 expression, however, was noted in cancers that originate from fibrous tissues, including gynaecological cancers, such as cervical (CESC), endometrial (UCEC), uterine (UCS) and ovarian (OV) cancers. The downregulation of FBN1 in this cohort of cancers may be suggestive of tissue-specific expression compared to up-regulation in other malignancies. Based on a previous study, FBN1 has a single CpG-rich dominant promoter that is highly conserved in mammals (29). Interestingly, a study showed that gene expression and activity of the promoter was significantly higher in MG63 cells (a human osteosarcoma line) when compared to MDA-MB-231 cells (a breast cancer cell line) (29). This agrees with previous observations that variations in the activity of the promoter region can exert a heritable transcriptional effect (30,31). As such, this might also explain, at least in part, the varying expression of FBN1 among different cancer types. Indeed, transcription factor binding motifs identified in the promoter region of FBN1, subserve tissue-specific functions (29). Of note, furin expression is slightly elevated in ovarian cancer compared to controls (data not shown). Therefore, in terms of the secretion of the cleaved peptide asprosin, the dynamics may be different. Moreover, it is possible that FBN1/asprosin may exert different effects in health and disease. For example, in the normal ovary, it might affect steroidogenesis and in the cancerous tissue may be implicated in the Warburg effect. Especially the later, warrants further investigation given that asprosin is a glucogenic peptide, that stimulates the release of glucose from hepatic cells. It is well known that in cancer cells, there is dramatic increase of the rate of glucose uptake and subsequent lactate production (32). Recently, inhibition of Bcl2 in SKOV3 ovarian cancer cells, appeared to reverse the Warburg effect and promoted oxidative stress-induced apoptosis *in vitro* (33). Further studies are needed to investigate the clinical application of asprosin as a potential mediator of the Warburg effect in ovarian cancer.

Furthermore, changes in the methylation status of FBN1 have shown biomarker value. For example, hypermethylation of Synuclein Alpha (SNCA) and FBN1 in stool samples show excellent sensitivity and specificity for colon cancer (34). Additional data has shown similar potential for colorectal cancer (26). The data presented in our study support these findings as methylation of FBN1 is significantly higher in colon adenocarcinoma (COAD) compared to healthy colon. Similar results are seen for breast (BRCA) and uterine endometrial carcinoma (UCEC). Methylation of FBN1 in normal ovarian tissue requires further investigation, since there is a lack of comparable normal methylation data held through TCGA and SMART for ovarian cancer.

*In silico* data for ovarian cancer were further validated using clinical ovarian tissue samples from patients with stage III/IV ovarian cancer, as well as both cancer and normal human ovarian epithelial cell lines Our present findings show significantly downregulated FBN1 expression in the ovarian cancer samples compared to those from healthy controls, in accord with the data noted in GEPIA. Moreover, FBN1 expression was detected in the human ovarian cancer cell line SKOV-3, the high-grade serous PEO1 and PEO4 cell lines, and the human endometroid ovarian cancer cell line MDAH-2774, as well as the normal ovarian epithelial cells (OSE). As noted in the clinical ovarian cancer samples, these human ovarian cancer cell lines exhibit relatively lower FBN1 expression compared with the normal ovarian tissue.

The differential - albeit not-significant - expression of FBN1 in the BRCA2 mutant and silent (wild-type) PEO1 and PEO4 cell lines, respectively, is of interest given that the tumour suppressor gene BRCA2 is an inhibitor of FBN1 (18). BRCA2 inhibition of FBN1 is associated with the inhibition of matrix metalloproteases (MMPs), including MMP2, MMP9 and MMP13, as well as the activation of cellular adhesion molecules which protect against metastasis (18). However, it should be noted that database analyses on survival times are often biased because of limited clinical information. These should be validated ideally with prospective cohorts employing sophisticated statistical considerations. Due to the nature of this study, such advanced statistical considerations were not feasible, thus this should be acknowledged as a limitation of the present study. The prognostic value of FBN1 in cancer is continually evolving (19,35,36). In our study, high FBN1 expression predicts lower overall- and progression-free survival of patients with late stages (i.e., III and IV) of ovarian cancer, corroborating previous findings which show promising prognostic potential of FBN1 as part of a panel of genes (19,35). Similarly, elevated FBN1 expression in both colon and bladder cancer are associated with worse overall survival (19,37,38).

Interestingly, previous data suggest that glucose metabolism including hyperglycaemia in ovarian cancer is associated with tumour growth and progression as well as worse survival outcome (20,39). As such, further research is required to elucidate the potential role of the glucogenic hormone and product of FBN1, asprosin, at the level of the ovaries. To that aim and following studies on the expression in normal murine and bovine ovaries (13,14), we provide novel data regarding the expression of both asprosin and its cognate receptor, OR4M1, in normal human ovaries and ovarian cancer.

Using immunohistochemical staining, we show aberrant protein expression of asprosin in ovarian cancer samples and normal adjacent tissue. In routine examination, normal adjacent tissue is often taken from the vicinity (<2 cm) of malignant cells and is frequently used as a control for cancer studies. Of note, recent transcriptome profiling data comparing normal adjacent tissue samples to healthy control tissue - which is removed from a substantial distance away from the primary tumour or from an age matched healthy control - suggest that there is premalignant conditioning of normal adjacent tissue (40). In the present study no apparent differences in asprosin protein expression were observed amongst different histological subtypes or stages of ovarian cancer, with staining representative of high asprosin expression in most cases (cancer and normal adjacent tissue samples). Similar widespread protein expression of asprosin was recently documented in malignant mesothelioma (41).

In our cell lines, this cytoplasmic distribution appeared associated with structures resembling microtubules or cytoskeleton. As this is a secreted protein, one would expect to observe a pattern that resembles the endoplasmic reticulum, or the Golgi, or even a vesicular pattern. One possibility is that asprosin production by furin-mediated cleavage, escapes the conventional secretory route, and follows a non-conventional secretory pathway that may not be dependent on vesicular exocytosis. Future *in vitro* studies using specific markers of cytoplasmic organelles should address this finding. One of the limitations of this study is the inability to measure mRNA expression for asprosin, as this is a cleaved peptide therefore only protein and precursor *FBN1* mRNA expression can be measured. The fact that FBN1 colocalises with furin in the ovary, favours local production of the cleaved product. Future studies should include the measure of asprosin levels from conditioned media of ovarian cell lines and/or ovarian explants to elucidate the secretion rate of asprosin from this tissue.

Moreover, given that asprosin binds to a GPCR, it is expected to have a discrepancy between mRNA and protein levels. It is possible after prolonged exposure to the ligand (i.e. asprosin), OR4M1 might undergo desensitisation, as a mechanism limiting GPCR signalling and subsequent activation of adenylyl cyclase. In doing so, OR4M1 can be detected in the cytoplasm (rather the cytoplasmic membrane) as a process of internalisation, or in lesser amounts if it undergoes lysosomal degradation rather than recycling (42). Future studies should concentrate on activation of second messengers *in vitro*. It is known that GPCRs are capable of activating multiple G proteins (43), therefore it is important to measure release of cAMP, or IP3 and activation of PKA or PKC *in vitro*. It has also been shown that asprosin is capable of binding to Toll-like receptor 4 (TLR4) and activating JNK mediated-pathway in pancreatic β-cells (44). Of note, the role of TLR4 in ovarian cancer is well documented (45), and future studies should also investigate the possibility of asprosin binding to TLR4 as well in ovarian cells.

We acknowledge that there are certain additional limitations in this study. The validation of the *in silico* data is performed on a small number of clinical samples and controls. Moreover, the samples of ovarian cancer where RT-qPCR was performed were all stage III/IV, as such we do not have the data to compare mRNA expression of FBN1 and OR4M1 in early stages of ovarian cancer. *In silico* analysis (Ualcan; http://ualcan.path.uab.edu) indicated that FBN1 is overexpressed across all stages with significantly increased expression when comparing stages II vs. III and II vs IV (data not shown). In addition, we demonstrate protein expression of asprosin in clinical samples and cells, however the study does not examine whether the ovaries are capable of secreting this peptide, since this was beyond the scope of the present paper. Future experiments using conditioned media from ovarian cell lines and/or ovarian explants are planned which will enable us to answer this question.

In conclusion, to the best of our knowledge, this is the first study to demonstrate expression of asprosin and its cognate receptor, OR4M1, in the human ovaries in health and cancer, focusing specifically on ovarian cancer. The presence of the recently identified orexigenic and glucogenic hormone asprosin (the cleaved product of profibrillin-1) in the human ovaries suggests a specific endocrine and/or auto/paracrine role for asprosin in human female reproduction. Indeed, the novel findings of the present study open two distinct lines of investigation: the potential role and effects of asprosin in normal ovaries in terms of fertility and steroidogenesis; as well as the potential involvement of asprosin as a gluconeogenic peptide in cancer. The latter is of particular importance given that hyperglycaemia is a contributing factor to the onset and progression of epithelial ovarian cancer (20). However, it should be noted that the exact role of asprosin and its receptor in the pathogenesis of ovarian cancer and its precise clinical relevance remains to be clarified. Further research is required to expand on the present findings and elucidate the potential role of asprosin in health and disease using *in vitro* and *in vivo* models, as well as larger cohorts of patients undergoing treatment for ovarian cancer.

## Supplementary Material

Supporting Data

Supporting Data

## Figures and Tables

**Table I.** Clinical details of patients with ovarian cancer.

Patient	Histology	Grade	Stage	Age, years
1	Serous	3	IIIC	64
2	Serous	3	IIIC	48
3	Serous	3	IIIC	61
4	Serous	2	IIIC	54
5	Serous	3	IIIC	69
6	Serous	3	IV	65
7	Serous	3	IIIC	75
8	Serous	3	IIIC	65
9	Serous	3	IIIC	56
10	Serous	3	IIIC	64
11	Serous	3	IIIC	64
12	Serous	2	IIIC	56

**Table II.** Clinical details of the control group.

Patient	Number of fibroids/patient	Mean diameter of fibroids/patient, cm	Age, years
1	1	8.9	39
2	4	3.2	42
3	2	6.5	40
4	6	3.7	43
5	2	6.0	45
6	1	10.0	41

**Table III.** Bio-Rad thermal cycling protocol for use with iTaq™ Universal SYBR′ Green Supermix (Bio-Rad Laboratories, Inc.).

Step	Temperature, °C	Time, sec	Cycle
Activation	95	30	1
Denaturation	95	5	38
Amplification	60	30
Melt curve analysis	60	Increments of 5	Infinite

**Table IV.** List of primers utilized in the present study.

Gene	Primer sequences (5′-3′)
YWHAZ	Forward: AGACGGAAGGTGCTGAGAAA
	Reverse: GAAGCATTGGGGATCAAGAA
FBN1	Forward: TTTAGCGTCCTACACGAGCC
	Reverse: CCATCCAGGGCAACAGTAAGC
OR4M1	Forward: TCTGTTAATGTCCTATGCCTTCC
	Reverse: AATGTGGGAATAGCAGGTGG

FBN1, fibrillin-1; OR4M1, olfactory receptor 4M1; YWHAZ, tyrosine 3-monooxygenase/tryptophan 5-monooxygenase activation protein ζ.

## Data Availability

The datasets used and/or analysed during the current study are available from the corresponding author on reasonable request.

## Abbreviations

ACC: adrenocortical carcinoma
BLCA: bladder urothelial carcinoma
BRCA: breast invasive carcinoma
CESC: cervical squamous cell carcinoma and endocervical adenocarcinoma
CHOL: cholangiocarcinoma
COAD: colon adenocarcinoma
DLBC: lymphoid neoplasm diffuse large B cell lymphoma
ESCA: oesophageal carcinoma
GBM: glioblastoma multiforme
HNSC: head and neck squamous cell carcinoma
KICH: kidney chromophobe
KIRC: kidney renal clear cell carcinoma
KIRP: kidney renal papillary cell carcinoma
LAML: acute myeloid leukaemia
LGG: brain lower grade glioma
LIHC: liver hepatocellular carcinoma
LUAD: lung adenocarcinoma
LUSC: lung squamous cell carcinoma
MESO: mesothelioma
OV: ovarian serous cyst-adenocarcinoma
PAAD: pancreatic adenocarcinoma
PCPG: pheochromocytoma and paraganglioma
PRAD: prostate adenocarcinoma
READ: rectum adenocarcinoma
SARC: sarcoma
SKCM: skin cutaneous melanoma
STAD: stomach adenocarcinoma
TGCT: testicular germ cell tumours
THCA: thyroid carcinoma
THYM: thymoma
UCEC: uterine corpus endometrial carcinoma
UCS: uterine carcinosarcoma
UVM: uveal melanoma
