# Supplementary material for: A pancancer overview of FBN1, asprosin and its cognate receptor OR4M1 with detailed expression profiling in ovarian cancer
Source: Oncol Lett. 2021 Jul 9;22(3):650. doi: 10.3892/ol.2021.12911 (PMC8298991; doi:10.3892/ol.2021.12911)

Figure S1. Methylation analyses of CpG island regions of FBN1 in The Cancer Genome Atlas cancer datasets taken from the Shiny Methylation Analysis Resource Tool. β values for FBN1 are shown for cancer (red) and normal (grey). Significant differences in the FBN1 methylation of BRCA, CESC, CHOL, COAD, KIRP, PRAD, READ, THCA and UCEC. Significant change in methylation indicated as: *P<0.05; **P<0.01; ****P<0.0001. It must be noted that control data are absent for: ACC, DLBC, KICH, LAML, LGG, MESO, OV, TGCT, USC and UVM. ACC, acute myeloid leukaemia; BRCA, Breast invasive carcinoma; CESC, cervical squamous cell carcinoma and endocervical adenocarcinoma; CHOL, cholangiocarcinoma; COAD, colon adenocarcinoma; DLBC, lymphoid neoplasm diffuse large B-cell lymphoma; FBN1, fibrillin-1; KICH, kidney chromophobe; KIRP, kidney renal papillary cell carcinoma; LAML, acute myeloid leukaemia; LGG, brain lower grade glioma; MESO, mesothelioma; ns, not significant; OV, ovarian serous cystadenocarcinoma; PRAD, prostate adenocarcinoma; READ, rectum adenocarcinoma; TGCT, testicular germ cell tumours; THCA, thyroid carcinoma; UCEC, uterine corpus endometrial carcinoma; USC, uterine carcinosarcoma; UVM, uveal melanoma.

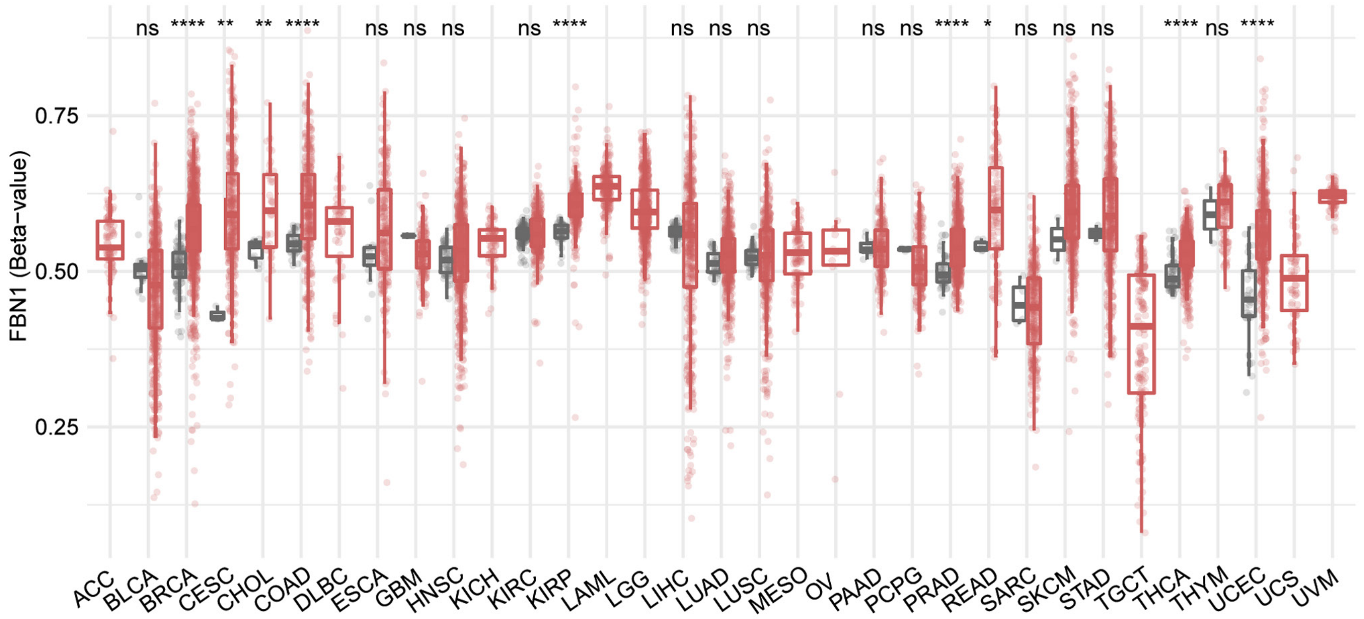

Supplement: Supporting Data [file Supplementary_Data2.pdf]
